# Assessment of the Irradiation Exposure of PET Film with Swift Heavy Ions Using the Interference-Free Transmission UV-Vis Transmission Spectra

**DOI:** 10.3390/polym13030358

**Published:** 2021-01-22

**Authors:** Adil Z. Tuleushev, Fiona E. Harrison, Artem L. Kozlovskiy, Maxim V. Zdorovets

**Affiliations:** 1 Flerov Laboratory of Nuclear Reactions, Joint Institute for Nuclear Research, 141980 Dubna, Moscow Region, Russia; tuleushev@jinr.ru; 2 Engineering Profile Laboratory, L.N. Gumilyov Eurasian National University, Nur-Sultan 010008, Kazakhstan; fiona_e_harrison@hotmail.com (F.E.H.); kozlovskiy.a@inp.kz (A.L.K.); 3 Laboratory of Solid State Physics, The Institute of Nuclear Physics, Almaty 050032, Kazakhstan; 4 Department of Intelligent Information Technologies, Ural Federal University, 620075 Yekaterinburg, Russia

**Keywords:** polyethylene terephthalate, interference-free transmission curves, red shift, ion irradiation induced light absorption, spiralization, extended conjugated systems, structure of deep traps in PET film

## Abstract

This paper presents the results of a study of polyethylene terephthalate (PET) films irradiated with Ar and Kr ions at both normal orientation and an angle of 40° to the normal. Normal irradiation was performed using Ar^8+^ and Kr^15+^ ions with an energy of 1.75 MeV/au and fluences in the range (2–500) × 10^10^ cm^−2^ for Ar^8+^ ions and (1.6 − 6.5) × 10^10^ cm^−2^ for Kr^15+^ ions. Kr ions with an energy of 1.2 MeV/au and charges of 13^+^, 14^+^, and 15^+^ were used for angled irradiation. For each Kr ion charge value, three fluence values were used: 5 × 10^10^, 1 × 10^11^, and 2.5 × 10^11^ cm^−2^. It is well known that irradiation of PET films by swift heavy ions results in a red shift of the UV-vis transmission spectra absorption edge. The experimental transmission spectra exhibit well-defined interference fringes, which obscure the underlying transmission response. Using an existing technique to obtain interference-free transmission curves *T_α_(λ)* for both pristine and irradiated PET film samples, we found that *S,* the total radiation-induced absorption of light by the PET film, is proportional to the logarithm of the fluence *F*. In addition to this dependence on the irradiating fluence, we also found that the charge of the irradiating ion has a significant influence on the position of the absorption edge in the UV-vis spectra. This provides experimentally independent evidence to confirm our previous results showing that ion charge has an effect on the post-irradiation state of PET films. We present a physical interpretation of the observed absorption edge red shift in irradiated PET films as being due to the growth of extended conjugated systems via the formation of intermolecular helical structures. Our investigations into the stability of irradiation-induced effects in PET films show that comparison of UV-vis transmission spectra before and after annealing can provide information about the structure of deep traps in PET.

## 1. Introduction

Experimental studies of the properties of polymer films irradiated with swift heavy ions are of interest to a large number of research groups: for reviews see for example [1,2]. Much recent attention has focused on the properties of polymer films irradiated with swift heavy ions without any subsequent etching. The potential for their industrial application has been demonstrated in a number of works [3,4,5,6,7], for example, ultrafast ion sieving and reduction of pollutants in industrial effluent. The growing interest in the practical use of the effects occurring in polyethylene terephthalate (PET) films irradiated with high fluences of swift heavy ions with an energy over 1 MeV/au without subsequent etching, as in [4], brings with it a growing interest in methods of reliably determining and controlling the irradiation exposure.

An analysis of a number of experimental works from the past 20 years suggests that there are two principal methodological approaches, although some authors (including ourselves in earlier papers) state fluence values without commenting on how these were determined [3,5,6,7,8,9,10,11,12,13,14,15,16,17,18]).

The first approach is associated with determining the intensity of the ion flux, and the second with determining the reaction of the polymer film to irradiation. Calibration of the ion current with a precision of ~20% using a Faraday cylinder and beam control by monitoring the signal from a secondary-electron emitting Al-foil detector placed in front of the samples was noted in [19,20,21]. In [22], the ion current during irradiation of polymer films was monitored by the characteristic secondary X-ray from a metal film located in front of the irradiated sample. Estimating the fluence from measurements of the current is significantly unreliable due to the instability of the ion beam itself and 20% precision is likely to be at or near the limit possible with such methods. While this is sufficient for research experiments [8,9,10,11,12,13,14,15,16,17,18], industrial applications typically require a higher level of accuracy, with no more than 10% statistical deviation from the average value. This can be achieved by direct counting of etched traces of latent tracks on the surface of irradiated films using scanning electron microscopy (SEM) [4,23,24,25,26,27]. According to [27], determining the irradiation exposure by counting the etched traces also has its limitations, since even when using high-resolution SEM, direct counting is possible only up to doses of (2–3) × 10^10^ cm^−2^. There is, however, considerable interest in higher irradiation fluences: in [4], where a linear dependence of the ionic transport rate on the irradiation exposure was found, the highest value of the transport rate was achieved at a fluence of 5 × 10^10^ cm^−2^. This, and higher, irradiation exposures are either very difficult or inaccessible to determine using SEM.

A number of authors [4,19,27,28,29] have shown that the absorption edge of the UV-Vis transmission spectra in a number of polymers, including PET, has a pronounced red shift with increasing irradiation fluence that depends on the type and fluence of the irradiating ions. In [19], samples of commercial polyimide (PI) films with thicknesses of 12, 25, and 50 µm irradiated with Ti, Mo, and Au ions with an energy of up to 11.4 MeV/au were studied. The absorption edge, located in the wavelength range of 420–550 nm for the pristine PI film, was found to spread to near-infrared wavelengths at high fluences. Taking the UV-Vis transmission spectra of a 12-µm thick PI film irradiated with Au ions with an energy of 5.2 MeV/au at fluences ranging from 1 × 10^10^ to 4 × 10^11^ cm^−2^ in the wavelength range 400–900 nm, the authors showed that the fluence can be characterized by the derivative *dT*/*dλ* of the transmission *T(λ)* and wavelength *λ.* By comparing the behaviour of this derivative for different fluences and for Au, Mo, and Ti ions, it was found that once above a certain value of irradiation exposure (of the order of 1MGy) the maximum value of *dT/dλ* shifts to longer wavelengths with a linear dependence on the fluence.

In [4], the UV-Vis spectra of PET Hostaphan^®^ and Lumirror^®^ films were measured in the range of 200–800 nm after irradiation with Bi ions with an energy of 1.4 GeV at fluences from 5 × 10^9^ to 5 × 10^10^ cm^−2^, and a red shift of the absorption edge was found. In addition, at a wavelength of 365 nm, a linear dependence of the absorption coefficient on irradiation exposure was observed. The absorption edge in pristine PET films lies in the range λ = 310 − 330 nm and is much sharper than in PI films. The PET absorption edge experiences a stronger red shift relative to its initial value than in PI, to wavelengths in the region of 700 nm when irradiated with swift heavy ions [4,20,27]. In [29] it was shown that the difference in absorbance between irradiated and pristine PI films at certain wavelengths depended linearly on the fluence at a constant energy loss *dE*/*dx*. In [30] the irradiation of polystyrene (PS) film stacks with Ar ions with an energy of 1.37 GeV and fluences in the range of 1.1 × 10^10^ to 5.5 × 10^12^ cm^−2^ was studied. It was found that the difference between absorbance before and after irradiation is roughly linear with fluence. In [31] the irradiation of polycarbonate film stacks with Ar ions with an energy of 1.37 GeV and fluences in the range of 1.1 × 10^10^ to 5.5 × 10^12^ cm^−2^ was studied. As in [30], the radiation induced changes in absorbance at a given wavelength followed a roughly linear relationship with fluence.

In [19] the linear relationship between the irradiation fluence and the derivative *dT*/*dλ* was obtained directly from the measured transmission *T(λ)* without any assumptions about the value of the reflection coefficient. In [4,21,29,30,31] a linear relationship was shown between the irradiating fluence and the absorption coefficient at certain wavelengths, calculated from the Bouguer-Beer-Lambert law [32]. In [4] the authors calculated the absorption coefficient taking into account the loss of light due to reflection, while in [29,30,31] there is no mention of whether, or how, these losses were taken into account. This suggests that the results in these papers provide a broad estimate of the relationship rather than an accurate quantitative determination.

For the purposes of determining the irradiating fluence via measurements of the optical response of the polymer film, we are looking for a way of measuring the total response to irradiation (i.e., over all wavelengths where there is a change in the UV-vis transmission curve after irradiation) in the most direct fashion, without the need for additional assumptions about the validity of calculated parameters such as reflectivity. The direct approach used in [19] is therefore more robust than first seeking to calculate the absorbance for each wavelength via the Bouguer-Beer-Lambert law and then integrate over *λ*, but we have been unable to find any information in the literature concerning measurement of the change in total absorption across the UV-vis wavelength range after irradiation of polymer films with swift heavy ions.

A particular challenge is the presence of interference bands in the observed optical transmission spectra of PI and PET. These contain well-defined interference fringes in the longer wavelength part of the spectrum, starting approximately at a wavelength of 500 nm for PI [19,29] and 370–380 nm for PET [27]. These transmission spectra would therefore more correctly be called spectro-interferograms. The interference fringes mean that even small amplitude changes in the transmission will lead to *dT*/*dλ* containing an appreciable wave-like component, posing a challenge to determining which of the local maxima is the actual maximum of *dT*/*dλ* as it shifts to longer wavelengths within the interference region.

Interference fringes nevertheless provide information about the state of the polymer films under study both before and after irradiation. For example, in [4], interference fringes on the UV-vis spectra were used to determine the refractive index of both pristine and irradiated PET films. The authors report that the value of the refractive index is in the range 1.54–1.65 and does not depend on the irradiation fluence, indicating that absorption rather than reflection is responsible for reducing the intensity of light passing through the irradiated films.

The problem of extracting the smooth transmission curve *T_α_(λ)* from the experimental transmission curve *T(λ)* in the presence of interference fringes has been solved in [33] in relation to thin silicon films on a transparent substrate. The only assumption made by the author when deriving formulae to determine optical parameters from transmission curves is that the functions *T_M_(λ)* and *T_m_(λ)* describing the positions of the observed interference maxima *T_M_* and minima *T_m_* on the spectral transmission curve *T(λ)* must be continuous functions of *λ*. From the assumption of continuity of these functions, it is possible to build *T_M_(λ)* and *T_m_(λ)* from the experimentally determined maxima and minima of interference fringes by successive parabolic interpolation between, respectively, three consecutive maxima or minima to create a continuous function linking all the observed maxima/minima. In [33], the author divided the full curve into four absorption regions: (1) transparent, where transparency is independent of wavelength; (2) weak, where the transparency drops by approximately 10% as the absorption coefficient *α* begins to affect transmission; (3) medium, where *α* has a noticeable effect, and transmission is reduced by up to 25%; and (4) strong, where transmission radically decreases under the influence of *α*, and the interference fringes disappear. For regions of weak and medium absorption, the author found that the value of *T_α_(λ)* can be obtained from Equation (1):

*T_α_(λ) = [T_M_(λ) T_m_(λ)]^1/2^* (1)

i.e., the value of *T_α_(λ)* is defined as the geometric mean of *T_M_(λ)* and *T_m_(λ).* It has been shown in [27] that in pristine and irradiated PET films, interference fringes occur in the experimental transmission curve *T(λ)* in a similar region of weak/medium transparency (up to about 15% reduction from maximum transmission). This suggests that the method of [33] can also be used to analyze the interference fringes in the transmission spectra from such polymer films to extract a smooth transmission curve *T_α_(λ)* from the experimental UV-vis transmission spectro-interferogram of polymer films irradiated with swift heavy ions.

It is well known that for swift heavy ions with an energy range of 1–10 MeV/au, the energy losses of the ions as they pass through the polymer film occur as a result of interaction with the electronic subsystem of the irradiated material [34]. It is also well known that the physical cause of the absorption edge in polymer films, including PET, is the internal photoelectric effect, in which photon absorption is explained by the redistribution of electrons over energy states in the irradiated film [35,36]. The observed red shift therefore reflects changes in the state of electrons in the film after irradiation, and the comparison of the smooth transmission curves *T_α_(λ)* for pristine and irradiated films over the entire wavelength range should provide quantitative information about the total irradiation fluence that the polymer film has been exposed to.

In the experiments reported here, we have used the method described in [33] to show that the smooth transmission curve provides a means of quantifying the total irradiation exposure of PET films. With appropriate initial calibration this offers a way to determine the irradiation fluence using only optical spectrometry data, which could also be used to create feedback to the ion source to control the irradiation process. We then consider the physical interpretation of these experimental results.

## 2. Experimental Techniques

**Figure 1:**
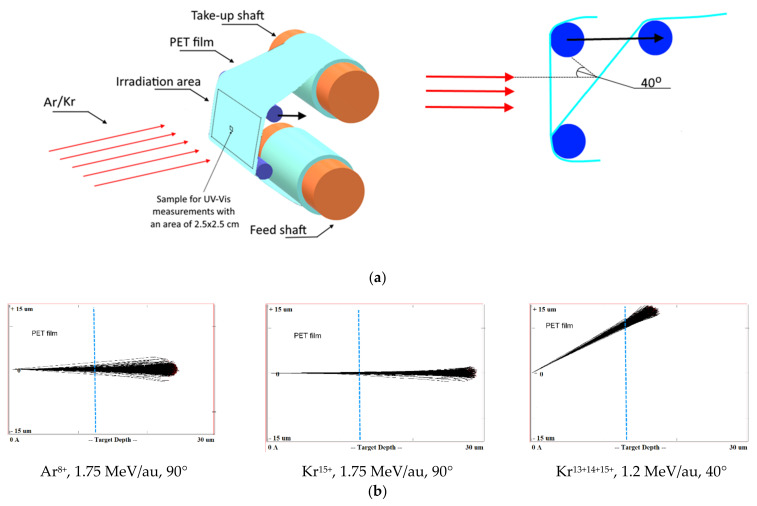

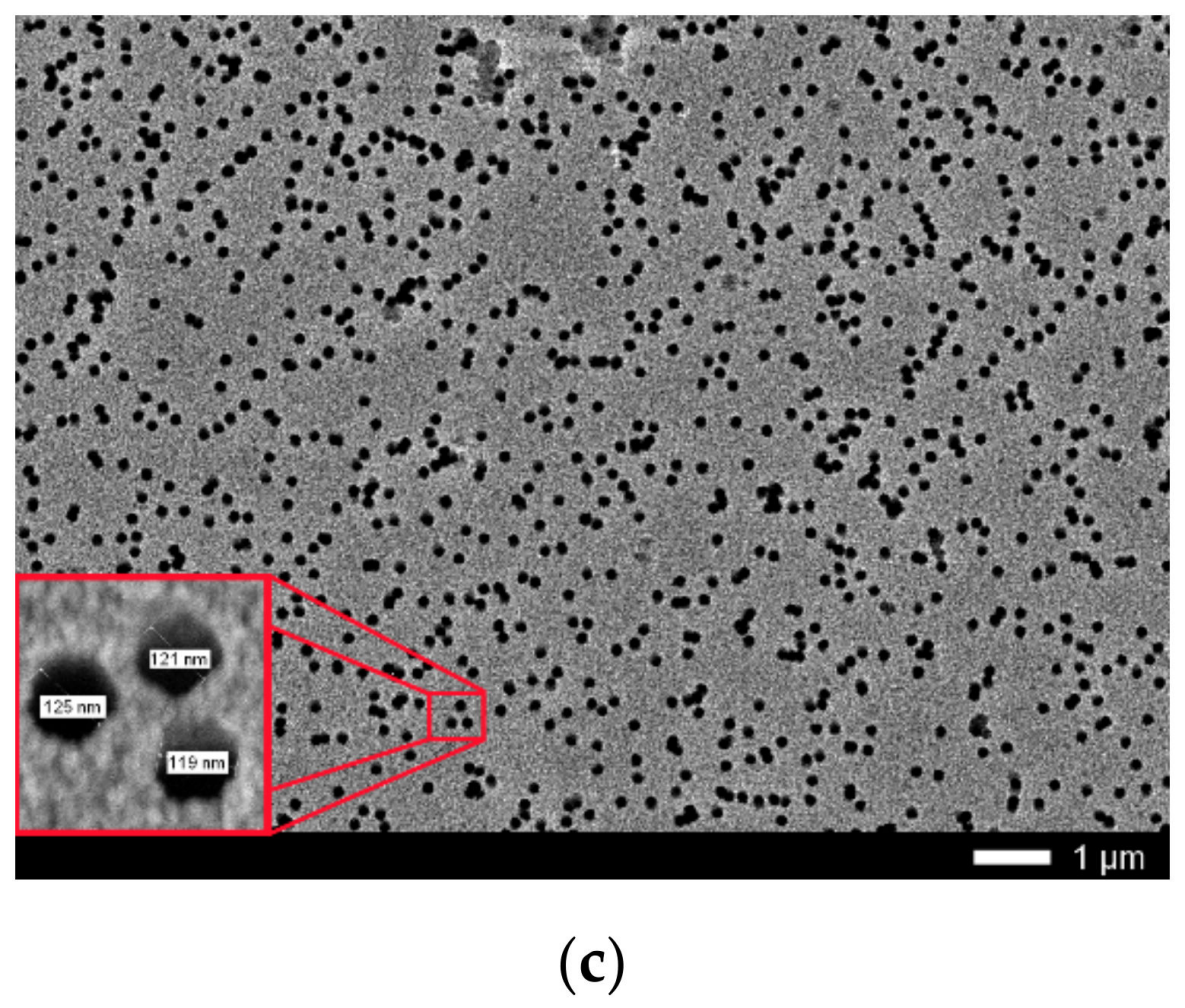
(**a**) Layout of film rolls for irradiation; (**b**) Screenshots SRIM projectile ion trajectories through polyethylene terephthalate (PET) film; (**c**) Scanning electron microscopy (SEM) image of calibrated PET film sample after chemical etching.

A PET roll film of the Hostaphan^®^ RNK-12 trademark manufactured by Mitsubishi (Hood Rd, Greer, SC, USA) with a nominal thickness of 12 µm and a width of 320 mm was irradiated at the DC-60 heavy ion accelerator in Nur-Sultan, Republic of Kazakhstan. The irradiation layout is shown in Figure 1a. Sections of the 300 × 200 mm film located between two support rolls were irradiated at normal orientation (i.e., the film vertical and ion beam horizontal in our set-up) and experiments repeated with the film tilted so that the ion beam was incident on the film at 40° to the normal, giving an ion path length of (12/cos40) ~16 µm. The irradiation angle was changed by shifting the upper roll relative to the lower one in the direction of the ion beam incidence, as shown by the arrow in Figure 1a. The deviation of the beam angle did not exceed 1° at the edges of the irradiation zone.

For normal exposure, ion fluxes of Ar^8+^ and Kr^15+^ with energy of 1.75 MeV/au and a flux of up to 2 × 10^8^ cm^−2^s^−1^ were used, as in [19,21,29]. Four irradiation fluences were used with Ar^8+^ ions: 2.25 × 10^10^ cm^−2^; 4.5 × 10^10^ cm^−2^; 6 × 10^11^ cm^−2^; and 5 × 10^12^ cm^−2^. For Kr^15+^ ions, three fluences of 1.6 × 10^10^ cm^−2^, 3.2 × 10^10^ cm^−2^ and 6.5 × 10^10^ cm^−2^ were used.

For irradiation at 40° angle, ^84^Kr ions with charges of 13^+^, 14^+^ and 15^+^ and of energy 100 MeV (1.2 MeV/au) were used. For each Kr ion charge value, three values of fluence were used: 5 × 10^10^ cm^−2^; 1 × 10^11^ cm^−2^ and 2.5 × 10^11^ cm^−2^. Table 1 shows the values of the magnetic field induction T and frequency of the high frequency generator HF at which the ^84^Kr ion beams were obtained for experimental use.

With these parameters for acceleration, beams of Kr ions with three charge values (13^+^, 14^+^ and 15^+^) at constant energy were obtained by the DC-60 accelerator. Using the SRIM Pro 2013 software [34] the estimated distance of travel of the Ar ions used for irradiation is about 22 µm, and those for the Kr ions with energies of 1.75 MeV/au and 1.2 MeV/au are about 27 µm and 20 µm, respectively, ensuring the through passage of bombarding ions in our experiments for both irradiation geometries. Figure 1b shows the screenshots of the SRIM projectile ion trajectories in PET film for normal exposure with Ar^8+^ and Kr^15+^ ions and angled exposure with Kr^15+14+13+^ ions calculated by the SRIM program, where the blue line marks the thickness of the film used in our experiment.

Widespread use of the TRIM/SRIM program code over more than two decades has shown that it predicts the ion path length with an accuracy of within a few percent for most ions, with the exception of the heaviest ions (such as Bi, Xe, Au, U) with high energies when passing through targets consisting of light elements [24]. A very recent study [27] has quantified the overestimation by TRIM/SRIM in path lengths for very heavy ions in light matrices as 10%. This finding does not apply to our situation, but even if the TRIM/SRIM results in Figure 1b overestimated the path length by 10%, the ions we have used quite clearly have sufficient energy to pass through the PET films in both irradiation geometries.

Calibration determination of irradiation exposure was performed by direct counting of etched traces of latent tracks on the surface of film samples irradiated up to a dose of 1.1 × 10^9^ cm^−2^ with Ar^8+^ and Kr^15+^ ions using a JEOL 7500F SEM (Akishima, Tokyo, Japan). The film was etched in a 2.2 M NaOH solution for 3 min at 80 °C, followed by washing and drying. Figure 1c shows the SEM image for the PET film used for calibration of irradiating fluence. Direct counting of the pores obtained after chemical etching using the MARKER-12 program showed that the pore density was 1.1 × 10^9^ cm^−2^. The average diameter of the through-etched pores was about 100 nm with an average inter-track distance of about 300 nm which made it possible to distinguish individual etched pores with confidence. Increased fluences were achieved by increasing the irradiation exposure time of PET film samples at a constant value of the ion current, which was periodically controlled using a Faraday cylinder. The accuracy of determining the fluence values was 10%.

The UV-vis optical properties of the irradiated films (including those used for calibration, before etching) were studied using the Jena Specord-250 BU analytical spectrophotometer (Analytik Jena, Jena, Germany). Transmission spectro-interferograms were recorded immediately after irradiation in the spectral range 280–750 nm, with a step of 1 nm for the films irradiated under normal exposure and with a step of 0.1 nm for those irradiated under 40° angle tilted irradiation. For spectral measurements, samples of 25 × 25 mm were cut from the central part of the irradiated films, marking the position relative to the roll to ensure a record was kept of the direction of the texture of the pristine film [16,37]. The accuracy with which *T_α_(λ)* can be determined is related to the accuracy of determining the positions of the interference maxima and minima *T_M_* and *T_m_*, which in turn is determined by the granularity of the measurement interval used in measuring the transmission spectrum and for a 1 nm step is about 0.1% [33].

The thermal stability of irradiation-induced effects was studied by annealing a PET film sample after irradiation at an angle of 40° with Kr^13+^ ions of energy of 1.2 MeV/au and a fluence of 5 × 10^10^ cm^−2^. The sample was placed in an air-evacuated sealed case made of non-irradiated PET film to prevent oxygen access. The sample prepared in this way was annealed in a SNOL drying cabinet (Snol-term, Tver, Russia) at a temperature of 75 ± 2 °C with no access to light for 160 hours.

Studies of structural changes in the irradiated PET films were performed by X-ray diffraction in the Bragg-Brentano geometry in the angular range of 2θ = 5 − 30° and azimuthal sweep φ = 0 – 2 π in increments of 10° using the D8 Advance Eco Bruker X-ray diffractometer (Bruker, Karlsruhe, Germany).

## 3. Experimental Results

**Figure 2:**
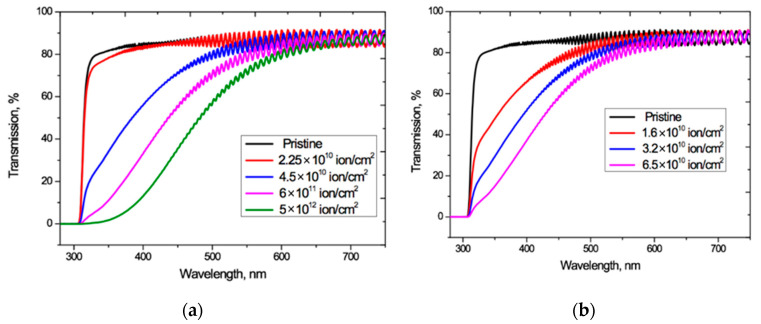
UV-vis spectro-interferograms of the PET films before and after normal exposure to various irradiation fluences for: (**a**) Ar^8+^ ions; and (**b**) Kr^15+^ ions.

Figure 2 shows the UV-Vis spectro-interferograms of the studied PET films before and after irradiation at normal incidence, for various fluences of Ar^8+^ and Kr^15+^ ions. Interference fringes are clearly manifest in the entire visible light range but are not present in the UV range.

For both Ar^8+^ and Kr^15+^ ions, as the fluence increases, the absorption edge shifts to longer wavelengths, and the wavelengths at which the interference fringes disappear also increases.

These interference fringes result from the coherent combination of reflected wave amplitudes (not intensities). The amplitude reflection coefficient is equal to *R^1/2^*, where *R* is the optical boundary reflection coefficient (film-air in our case), so even small values of the reflection coefficient in the regions of high transmission lead to noticeable interference effects in the spectral transmission function *T*(*λ*) [38]. At normal incidence, the reflection coefficient *R* is related to the refractive index *n_1_* of the incoming medium and the refractive index *n_2_* of the medium into which wave passes by the Fresnel formula *R = (n_2_—n_1_)^2^*/*(n_2_+n_1_)^2^* [4,39]. For an untreated PET film, a value of *n* = 1.57 − 1.58 is reported in [40] (although without specifying the spectral range of measurements), which is close to the value of 1.54–1.65 reported in [4] and gives an estimate for *R* at the film/air boundary of about 0.05.

Interference patterns can be characterized numerically by the fringe visibility parameter *V*, which is defined in Equation (2) as:

*V = (I_max_ − I_min_)/(I_max_ + I_min_)* (2)

where *I_max_* and *I_min_* are respectively the values of light intensity in the bright and dark fringe sections of the interference pattern [39]. In the absence of absorption in the film, a reflection coefficient of 0.05 should lead to the formation of interference fringes with visibility of up to 10%.

**Figure 3:**
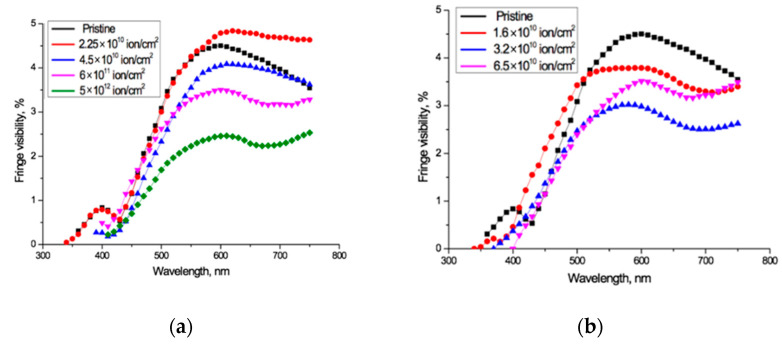
Visibility of interference fringes for the spectro-interferograms shown in Figure 2 for: (**a**) Ar^8+^ ions; and (**b**) Kr^15+^ ions.

The experimentally measured fringe visibility patterns for the spectro-interferograms in Figure 2 are shown in Figure 3. Observed visibility is 3–5% in the red wavelength range of the spectrum for all the samples under study. At wavelengths below 600–550 nm, the visibility of all interference patterns decreases rapidly in a very similar way, to less than 1% below about 450 nm. The fact that the observed fringe visibility is lower than the calculated estimate shows that there is some absorption within the PET film, which is thick compared to the wavelengths of the interfering light. The relatively small differences between observed visibilities for all fluences shows that the reflection coefficient has no significant dependence on the fluence, which broadly confirms the conclusion of the authors in [4] that decreases in the intensity of light passing through irradiated films are due in the main to increased absorption. The fact that the fringe visibility patterns in Figure 3 show a clear dependence on wavelength *λ* demonstrates that the amplitude reflection coefficient, and therefore the optical boundary reflection coefficient, are also functions of the wavelength *λ*. This dependence needs to be taken into account when making quantitative calculations based on experimental UV-Vis spectra.

**Figure 4:**
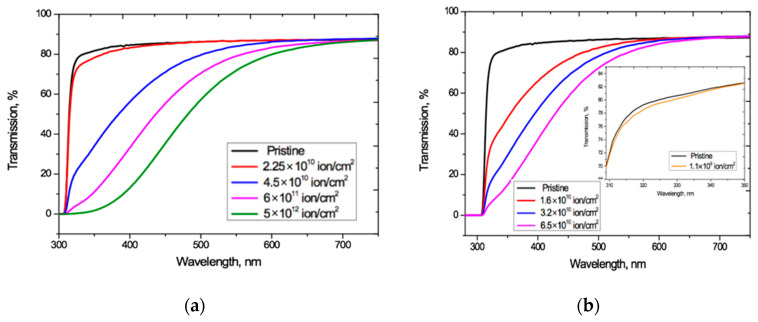
Interference-free transmission curves *T_α_*(*λ*) of the irradiated PET films obtained from Figure 2 for: (**a**) Ar^8+^ ions; and (**b**) Kr^15+^ ions.

Given these observations, we used the relation proposed in [33] to determine *T_α_*(*λ*) over the entire wavelength range where interference fringes exist, including the zone of weakly manifested fringes since, being so small, they will have negligible effect on the final shape of *T_α_*(*λ*). The interference-free transmission curves *T_α_*(*λ*) obtained from applying the method of [33] to the spectro-interferograms in Figure 2, are shown in Figure 4.

Figure 4 shows that increasing the irradiation fluence leads to a monotonically increasing red shift of the PET absorption edge up to the visible light boundary, which correlates well with the results in [4,20,27]. Noting that the Ar^8+^ ion fluences we have used range up to two orders of magnitude higher than those used for Kr^15+^, we see that, at comparable fluences, irradiation with Kr^15+^ ions leads to a stronger shift of the absorption edge than with Ar^8+^ ions. For both types of ion and at all fluences, *F* has a weak effect on transmission at longer wavelength, where *T_α_*(*λ*) is also only weakly dependent on *λ* and has a value of 87.1–88%. This is very close to the value of 87.3% for pristine film, and also aligns with the conclusion in [4] that decreases in transmission through irradiated films are due to increased absorption.

The irradiation fluence of 1.1 × 10^9^ cm^−2^ used for calibration produced no changes in the shape of interference-free transmission curve *T_α_*(*λ*) for Ar^8+^ ions (so has not been plotted in Figure 4a as it coincides with that for pristine PET). For Kr^15+^ ions this calibration fluence led to a slight decrease in *T_α_*(*λ*) relative to the pristine curve over the wavelength range 310–350 nm, as shown in the inset in Figure 4b. This experimental result suggests there could be a fluence threshold beneath which irradiation with swift heavy ions produces no red shift of the absorption edge in PET film. We intend to investigate this in more detail through other experiments in the future.

In studying this set of interference-free transmission curves *T_α_*(*λ*) for irradiated and pristine PET films in the range *λ* = 300 − 750 nm, we have found that, *S*, the total radiation-induced absorption of light by the PET film, is proportional to the logarithm of the fluence *F* across a wide range of fluence values. We obtained this result via two steps. We first subtracted the pristine transmission curve *T_α_^P^*(*λ*) from each of the irradiated transmission curves *T_α_*(*λ*) to produce a (negative) transmission difference function.

**Figure 5:**
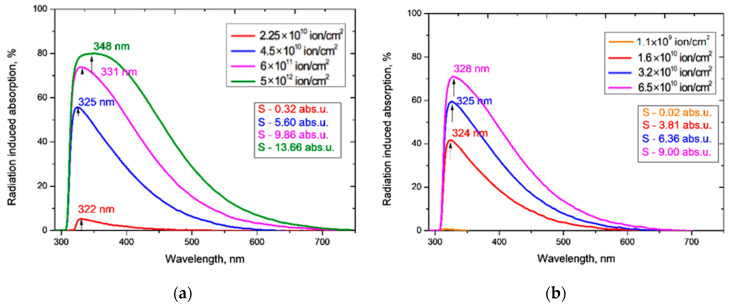
Radiation-induced absorption of the PET films obtained from Figure 4 for: (**a**) Ar^8+^ ions; and (**b**) Kr^15+^ ions.

The principle of the conservation of energy requires that the sum of transmission, reflection and absorption intensities equals the intensity of the incident light. Normalizing by the incident intensity, it follows that the difference in transmittance between an irradiated film and the pristine film is equal in magnitude and opposite in sign to the sum of the difference in absorption and reflection. As shown in [4], and supported by our results above (Figure 3), irradiation by heavy ions has a negligible effect on the reflection from PET films, so there is negligible reflection contribution to the difference function, and the (negative) differences in transmission are caused by (positive) differences in the absorption of light by the pristine and irradiated PET films. The difference curves in Figure 5 therefore show the total additional absorption induced by the irradiation fluence.

In order to avoid any misunderstanding, we note that the term absorption refers to the physical processes by which matter absorbs a photon’s energy, typically via its atomic electrons [35,36,41].

The term absorbance is defined as the logarithm of the ratio of incident to transmitted radiant power through a sample [42].

For collimated monochromatic incident radiation with a spectral bandwidth that is narrow compared to the spectral linewidths in the spectrum and homogeneous isotropic media, the absorbance is given by the Bouguer-Beer–Lambert law and is proportional to the absorption path length, *l*, and to the concentration, *c*, of the absorbing species [32]. The Bouguer-Beer–Lambert law only holds for monochromatic spectrally narrow radiation, where it permits the spectral absorbance (that is, the absorbance at a specific wavelength) to be calculated. It is used in [4,21,30,31] to determine the irradiation-induced spectral absorbance at certain wavelengths. Measurements of absorbance are widely used to characterize optical responses of irradiated polymer films at specific wavelengths.

We, however, are seeking to determine the total irradiation-induced absorption. To do so via such measurements and the use of the Bouguer-Beer–Lambert law, it would be necessary to find and integrate over the values of spectral absorbance at all wavelengths where there are changes in the intensity of the light transmitted through the polymer after irradiation. We have found no indication in the literature how this might be accomplished. Our method of subtracting the pristine transmission curve from each of the irradiated transmission curves is a more direct way to determine the total irradiation-induced absorption in PET films, that bypasses the need to calculate individual spectral absorbances across all wavelengths. We note that our method works for PET, since there is negligible irradiation-induced change in reflectivity so that it drops out of the difference equations. This would not be the case if irradiation did cause significant changes in reflectivity, as it might do in other materials.

**Figure 6:**
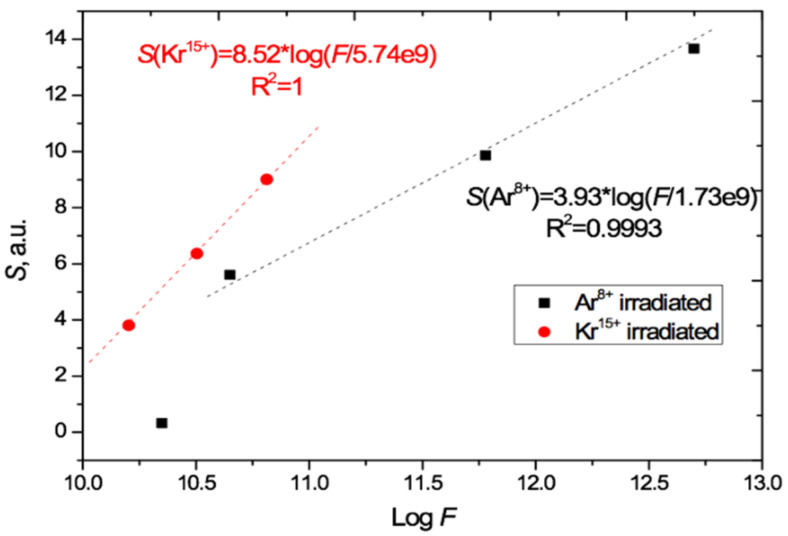
Relation of the induced total light absorption *S* to log*F* as calculated from Figure 5 for the PET films irradiated at normal incidence with Ar^8+^ and Kr^15+^ ions.

The total irradiation-induced absorption is given by the areas *S* under the radiation-induced absorption difference curves in Figure 5. The value of *S* has a good linear relationship to the logarithm of the irradiation exposure, for fluences *F* in the range (4.5 × 10^10^ – 5 × 10^12^) cm^−2^ when irradiated with Ar^8+^ ions and in the range (1.6–6.5) × 10^10^ cm^−2^ when irradiated with Kr^15+^ ions, as shown in Figure 6. We are measuring total absorption across a wide range of wavelengths rather than spectral absorbance at a specific wavelength. As with other integral/differential parameter pairs, there is no particular tension between our experimental results and measurements of the relationship between spectral absorbance and fluence, such as [4,21,29,30,31] which found a linear dependence between spectral absorbance and irradiating fluence at specific single wavelengths.

Comparison of the gradients of the two *S*/log*F* lines in Figure 6 shows that the effect of Kr^15+^ ions on light absorption is 2.2 times [8.52/3.93] stronger than Ar^8+^ ions in the range of applicability of this relationship. This is approximately 10% higher than the ratio of energy losses of these ions in PET film given by the LISE++ software (90.5 MeV for Kr and 44.5 MeV for Ar) [43].

In order to understand the stability of this relationship, we conducted the same experiments and analysis with a different ion path length, by changing the incident angle of irradiation to 40° to the normal and using three different ion charges Kr^13+14+15+^, all at the lower energy of 1.2 MeV/au. For each ion charge value, three values of fluence were used: 5 × 10^10^ cm^−2^; 1 × 10^11^ cm^−2^; and 2.5 × 10^11^ cm^−2^.

**Figure 7:**
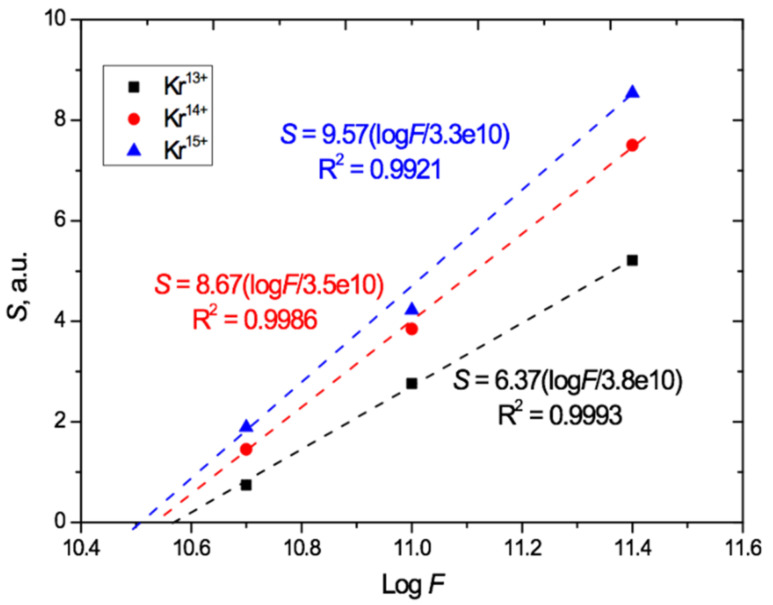
Relation of induced total light absorption *S* to log*F* for PET films irradiated at an angle of 40° with Kr^13+14+15+^ ions with an energy of 1.2 MeV/au.

The UV-vis transmission spectro-interferograms were processed in the same way as described above to remove the interference fringes. We found that the linear relationship between the total irradiation-induced absorption and the logarithm of the irradiation fluence is preserved for each of the Kr charges used (13^+^, 14^+^, and 15^+^), as shown in Figure 7. The data points show *S* values for the ion charges and fluences we used, and the dashed lines indicate the linear approximations based on the experimental results obtained.

Figure 7 shows that, at the highest fluence of 2.5 × 10^11^ cm^−2^, an increase in the charge from 13^+^ to 14^+^ led to an increase of 40% in the total absorption due to a larger red shift. When the charge was increased further to 15^+^, this increase was 60%. For the lowest irradiation exposure of 5 × 10^10^ cm^−2^, the relative increase in the total absorption with increasing ion charge is even more pronounced: equal to 1.7 and 2.2 times, respectively. This indicates a significant influence of not only the irradiation fluence but also the heavy ion charge on the position of the absorption edge in the UV-vis spectra of PET film. Despite this, as is well known, the ion charge is not an input parameter for estimating the energy loss of swift heavy ions in the LISE++ and SRIM software.

The extrapolation of the lines in Figure 6 and Figure 7 beyond the experimental points to intersect with the log*F* axis provides a very rough first estimate of the lower bound of the range of applicability of this logarithmic relationship, which clearly cannot hold for *S* < 0. For normal irradiation, this estimated lower bound is about 2 × 10^9^ cm^−2^ for Ar^8+^ and 6 × 10^9^ cm^−2^ for Kr^15+^ ions, and for angled irradiation with Kr^13+14+15+^ ions, the estimated lower bound on applicability is in the range (3–4) × 10^10^ cm^−2^.

The fact that for Ar^8+^ the logarithmic relationship already no longer holds at a fluence of 2.25 × 10^10^ cm^−2^ (the lowest Ar^8+^ data point in Figure 6), together with our observation of small effects in the transmission curve for the Kr^15+^ calibration fluence of 1.1 × 10^9^ cm^−2^ (see above Figure 4b and Figure 5b), points to the need for more detailed studies of PET films under low irradiation fluences. Such experiments could provide further insights into the emergence of the red shift of the absorption edge and better estimates of the lower bounds of applicability of this method of determining irradiation fluences by their logarithmic relation to *S.*

**Figure 8:**
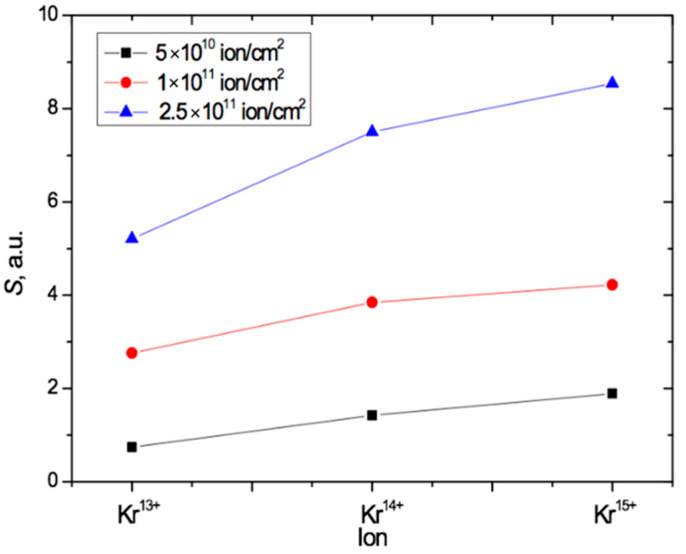
Plot summarizing the results in Figure 7, showing the dependence of *S* on both the fluence and the value of the ion charge for PET irradiated at an angle of 40° with Kr ions.

The results above are consistent with the conclusions in [18,44] and provide independent experimental evidence to that in [18] for the impact of ion charge on the post-irradiation state of PET films. Figure 8 summarizes our results for angled irradiation by plotting the values of the total radiation-induced absorption of light *S* for each of the three different Kr ion charges and the three different irradiation fluences. This shows that *S* increases both with increasing fluence and with increasing ion charge in the PET films in this study.

## 4. Interpretation of Results

The cause of the red shift of the absorption edge in irradiated transparent polymer films is generally considered to be due to a process of carbonization of polymer molecules under ion irradiation. This is indicated, for example, in the article [4] with reference to the article [19], which, in turn, refers the reader to earlier articles [30,45]. In seeking to understand the charge dependence of the red shift of the absorption edge that we have observed, we will begin with a more detailed analysis of the explanations in these articles.

In [45], studies of PS films with a thickness of about 300 nm irradiated with 300 KeV Ar^+^ and 100 keV He^+^ ions with a current density of up to 200 nAcm^−2^ and ion fluences of 5 × 10^13^ to 5 × 10^16^ cm^−2^ were reported. The red shift of the absorption edge in the UV-vis spectra of irradiated films was observed to increase as the fluence increased. This was attributed to the transformation of the polymer film into amorphous hydrogenated carbon (*a-C:H*) under irradiation, because the shape of the absorption edge corresponded to that of the fundamental absorption edge in *a-C:H.* The fluences used in [45] are very significantly higher (at least two to four orders of magnitude) than those used in the other experiments described in the introduction and in our experiments. Neither the ion charges nor the ion beam intensities are specified in [45] but for any usual Ar^+^ and He^+^ ion charges, these fluences correspond to ion beam fluxes of the order of 10^11^ cm^−2^s^−1^ and higher. These are also very significantly higher than the intensity of ion beams used in other experiments, such as [19,21,29], where fluxes of the order of 10^8^ cm^−2^ s^−1^ were used.

It is well known that the proportions of electronic and nuclear losses in the interactions between swift heavy ions and polymer films vary with the energy of the irradiating ions. Application of the SRIM program to the ion energy experimental conditions in [45] shows that they are characterized by approximately comparable proportions of electronic and nuclear losses. Moreover, a similar experiment in which PI films were irradiated with N_2_^+^ ions with energies of 0.3, 0.6, and 1.0 MeV, up to fluences of 10^14^–10^17^ cm^−2^ [46], showed that the temperature of the samples during irradiation was 300–400 °C. Such high levels of ion-nuclear interaction and high temperatures could very plausibly lead to carbonization of the irradiated PS film under the experimental conditions described in [45]. In the energy ranges studied in [4,19,20,27] and our own studies, however, nuclear losses are negligible compared to electronic ones.

We conclude that the explanation of the absorption edge red shift in [45] cannot be extended to the very different ion energy regime studied by ourselves and the authors cited above. We also note that red shifts of the absorption edge can occur without carbonization, for example in non-organic films: in [47] the shift is found in thin amorphous films of As_2_Se_3_ and Se, and in crystalline PbI_2_ when irradiated with He^+^ and Ar^+^ ions.

In [48], the effects in the latent tracks in PI films were studied after irradiation in air with Ar^+15^ ions of energy 22.5 MeV/au up to fluences of 8 × 10^11^ cm^−2^. The authors observed a change in the color of the films under irradiation, attributed to the loss of oxygen and nitrogen with simultaneous carbon enrichment. The focus of the discussion in this study was the observation of new radiation-induced peaks on the transmission curve and their behavior under changing fluences, rather than the red shift of the absorption edge itself. In [30], the authors observed an increasing shift of the absorption edge from UV to visible light as irradiation exposure increased and, with reference to [45], attributed this to carbonization of the material under irradiation, specifying that this process apparently occurs in the central part of the latent tracks. In [19] the darkening of color in the PI films is described as moving from amber to “dark graphite” suggesting that the color change is due to carbonization. Color changes are not, however, a reliable indicator of carbonization. Any body (including a polymer film) with any absorption mechanism will look black if it is strongly absorbing: blackening does not necessarily have to be associated with carbonization.

Likewise, an increased yellowing of the color [20] of PET film under irradiation does not imply carbonization. Yellow and purple-blue are complementary colors [49,50,51,52], and so the depletion of the UV-blue short wavelength components of the transmission spectrum of the initially transparent PET film will lead to yellowing, varying in intensity depending on the degree of depletion of the transmitted light. Yellowing of the irradiated film shows that the absorption edge is red-shifting away from UV towards longer wavelengths, but does not explain its cause.

From this review of previous work, it is apparent that there is no satisfactory explanation of the red shift effect in polymer films under the influence of swift heavy ion irradiation for the experimental conditions used by ourselves (and many others). The accepted carbonization explanation has a reliable base only in the experiments in [45], which were performed in a very different ion energy regime with a significant nuclear component of interaction, unlike the regime in our experiments where electron interactions dominate.

We consider that in PET films in the regime we are studying the observed effects can be explained on the basis of existing knowledge about the interaction of ions with matter and the molecular structure of polymers. We start from the fact that delta electrons, to which all the observed effects in the irradiated polymer films are attributed, are not the only electrons involved in these interactions. In their seminal work [53], Bethe and Ashkin state: “An energetic heavy charged particle, in its passage through the matter, produces the ionization contributing to its energy loss in two ways. In the primary collision with the electrons in an atom, the most probable of the ionizing collisions are those in which a relatively slow secondary electron is ejected with kinetic energy smaller than the ionization potential. A smaller fraction of ionizing collisions, however, produce secondary electrons of relatively high energy …so-called delta-rays”. When a charged ion passes through a polymer film, therefore, the final electron density in the core around the axis of the latent track will be determined by the ratio of delta electrons leaving this zone (and being lost in the track’s peripheral outer shell) to slow electrons being drawn into the core by the field of the passing ion. Since the delta electrons comprise the smaller fraction, we can reasonably expect that the contribution of the slow electrons to the final charge redistribution will dominate. The resultant excess electron density in the track core, and electron depletion of the peripheral outer shell, and the corresponding redistribution over energy levels, should be detectable in the UV-vis transmission spectra of the irradiated film.

The data accumulated after the discovery of the electret effect in polymer films in the 1960 s, including PET, and the widespread use of polymer electrets for commercial purposes, provides an understanding of the underlying physical processes here. The existing explanation of the behavior of polymers with respect to their electret properties is based on the use of a modified model of energy zones. According to this model, charges in polymers may be stored in both the electret volume (deep traps) and in shallow surface traps. Surface traps (delocalized states with energies from 0.02 to 0.87 eV) are associated with chemical inclusions and impurities, specific surface defects, broken bonds, adsorbed molecules, and differences between the surface and volume material. Deep traps (localized states with energies greater than 2 eV) are associated with three levels of structural charge capture. The first level is located in groups of atoms (for example aromatic groups) in molecular chains, the second level is located between groups of atoms in neighboring molecules, and the third is associated with crystalline regions or crystalline-amorphous boundaries. Since PET is a semi-crystalline material, these traps can be different in different molecular regions of the material and their depths can vary accordingly. In electret materials, the participation of shallow traps in charge capture and charge storage is small compared to the contribution of deep traps, but shallow traps play a critical role in charge transfer [54].

For deep traps in PET, values of photoactivation energies of about 4 eV or more have been found. Traps with these excitation energies are the principal ones for the accumulation and long-term retention of additional electrons during the formation of electrets. They are associated with aromatic groups in the polymer film and are responsible for the experimentally observed zone of strong light absorption in the near-UV region with an absorption edge at wavelengths greater than 300 nm [35]. From experiments on the photoconductivity of Lumirror^®^ PET films the authors also found that, as a result of the internal photoelectric effect, a photocurrent occurs at wavelengths ≤320 nm, with electrons being the dominant carriers. It was also found in [35] that the absorption edge observed in the transmission spectrum of a pristine PET film is already the result of a red shift of the absorption peaks of π–π* transitions in the benzene rings of repeat units of the PET chain molecule. The shift is caused by the resonance of the π orbitals with the two carbonyl groups directly attached to the benzene ring forming an extended conjugated system that stabilizes delocalized extra electrons inside it and accounts for the strong and stable electret effect in PET. In addition, traps with a depth of 2.85 eV [55] and 2.3 eV [56] have been found.

The various studies that have been conducted are in good agreement with respect to data on deep traps, as is to be expected since these depend on the molecular structure of the polymer. For example, Figure 5 shows clearly that almost all radiation-induced absorption in PET films occurs in the energy range of 2 to 4 eV (600–300 nm). This suggests that the upper energy of slow electrons responsible for the irradiation-induced red shift is about 4 eV. Data on the shallow traps show less agreement, which can be understood as reflecting the differences in technological additives in the PET films from different manufacturers. Nevertheless, it is believed that traps with an activation energy of 0.74 eV are associated with relaxation vibrations of carboxyl groups [57], and traps of 0.23–0.5 eV are associated with the movement of ester groups [58,59]. The activation energy of charge carrier mobility in PET, which according to [60] is about 0.3 eV, falls within the same range.

We now turn our attention to two important questions: how the electric field produced in PET by a swift heavy ion compares with those used to produce and research electrets; and how far from the ion latent track axis does this field remain strong enough to displace slow electrons in the direction of the axis (i.e., is sufficient to provide the electron mobility activation energy of 0.3 eV), which provides a measure of the diameter of the latent track.

We can estimate the electric field strength as follows. Using [4,40] to estimate the refractive index *n* of the PET film as *n* = 1.6, the relationship *ε = n^2^* [61] between *n* and the permittivity *ε* gives a value of *ε* = 2.5 for the PET film. For an ion with a charge of +15 and a Lorentz factor of ~10^−2^, we can use Coulomb’s law to estimate the electric field strength as being 25 V/µm at a distance of 20 nm from the track axis, which is the field strength used in [62] in the formation of electret PET films. For the initial part of the ion path (i.e., before it loses significant charge), the diameter of the region around the latent track axis where slow electrons are displaced towards the axis is about 100 nm. As a value for the diameter of the latent track, this correlates quite well with both the reported data obtained on the basis of measuring the radial etching rates of latent tracks [24] and our previous X-ray estimations [18].

Statistically, the onset of overlapping of latent tracks begins with the overlapping of the peripheral outer shells of the tracks, which are electron depleted. A track which overlaps an existing one will therefore have a lower electron density. As the extent of overlapping increases, there is an increasing likelihood that the peripheral outer shell of a new track will overlap with the electron-rich inner core of an existing one. This will not, however, make more electrons available to be captured in the new track, because these inner core electrons are held in deep traps. Since the probability of the overlapping of latent tracks increases with increasing ion fluence, later ions will have a progressively weaker effect on the electronic subsystems of the polymer film. The observed logarithmic relationship between the fluence and the radiation-induced total optical response of the PET film confirms this.

Formally, the experimental relationship between the fluence *F* and the total optical absorption *S* can be expressed by the equation:

*log(F*/*F_0_*) = *NS* (3)

where *N* and *F_0_* are constants. This equation is a solution of the differential equation:

*dS* = (log *e*/*NF*)*dF* (4)

which shows that the change in the total light absorption *dS* is inversely proportional to *F.* In other words, the longer the irradiation exposure continues, the smaller the additional change in the total light absorption of the irradiated PET film becomes. This reduced response indicates a decrease in the energy loss of the swift heavy ions passing through the film as the fluence increases.

**Figure 9:**
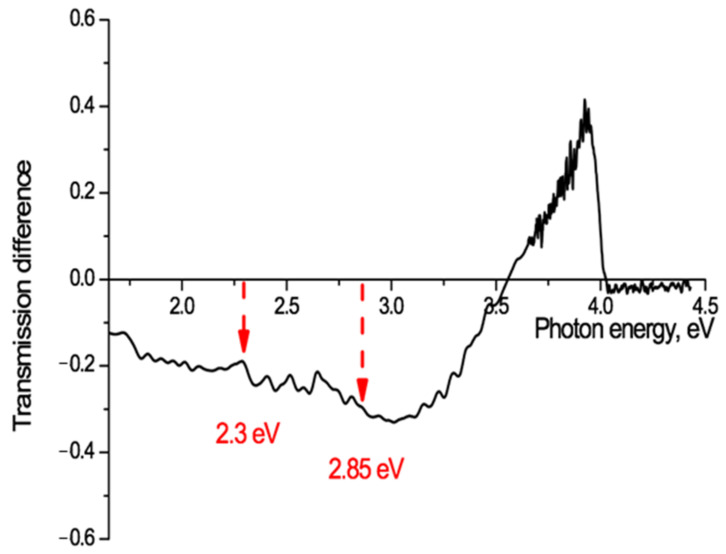
Transmission difference spectrum, before and after annealing for 160 hours at a temperature of 75 °C, for PET film irradiated at an angle of 40° with Kr^13+^ ions of energy 1.2 MeV/a.u and fluence of 5 × 10^10^ cm^−2^.

Electret studies commonly make use of thermally stimulated discharge (annealing) to investigate the release of charges from traps in pre-charged electret films [54]. Thermal stimulation causes metastable states to move towards their state of thermodynamic equilibrium. In our experimental situation it should provide information on where slow electrons have been trapped after ion irradiation and where they move to when de-trapped by heating. A sample of PET film, irradiated at an angle of 40° with Kr^13+^ ions of energy 1.2 MeV/au and fluence of 5 × 10^10^ cm^−2^, was annealed for 160 h at a temperature below the glass transition temperature and with no access to air and light, to stimulate thermal discharge of charges from traps. The UV-vis transmission spectrum after annealing was compared with that from before, and the difference spectrum for this sample is shown in Figure 9.

This shows a marked increase in the film transparency after annealing at photon energies of 3.5–4 eV (350–310 nm) with a sharp peak at just below 4 eV (313 nm), as a result of thermally stimulated charge de-trapping. There is also a decrease in transparency at lower photon energies (<3 eV) with a number of small local peaks.

The only traps in PET chain molecules with an energy of 4 eV are those associated with benzene-carboxyl complexes. Figure 9 provides clear confirmation that slow electrons are captured in the deepest molecular traps in the core of the latent tracks. It also confirms the existence of an inner electron-enriched core and peripheral electron-depleted zone in the latent tracks. The point at which there is no change of transmission (~3.6 eV in Figure 9) can be taken as an indicator of the border between these two regions in the latent tracks. Using our above estimation of *ε* = 2.5, for the initial part of the ion path we can estimate the radius of the boundary of the inner core to be about 4 nm. Different irradiating ion charges will have different electric fields and so lead to different distributions of electrons in their latent tracks and different estimates of the radii of the core and peripheral zones. This provides a qualitative understanding for the observed dependence on ion charge of the red shift of the absorption edge in PET films.

The observed redistribution of electrons, released by heating from the deepest molecular traps in the small volume electron-enriched core and moving to shallower traps in the much larger volume electron-depleted peripheral zone, suggests that this method of ion irradiation and annealing offers a novel means of studying the spectrum of traps in polymer films. Both of the known PET traps at 2.3 eV [56] and 2.85 eV [55] can be seen in Figure 9, which shows a well-defined peak of absorption growth between 2.22–2.36 eV with a maximum at 2.3 eV, and a fainter one at 2.84–2.89 eV with a maximum at 2.86 eV. The spectrum of absorption fringes in Figure 9 is, however, significantly richer and shows, for example, the presence of three more absorption peaks between 2.3 eV and 2.7 eV and one at 1.7–1.8 eV with a maximum at 1.73 eV, none of which, as far as we can establish, have previously been observed. A detailed discussion of the structure of the traps available for electron capture in the PET film is not the subject of this article, but we intend to devote a separate study to this interesting and important matter.

**Figure 10:**
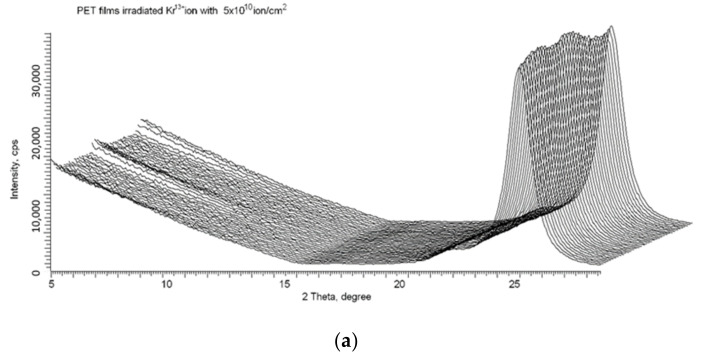

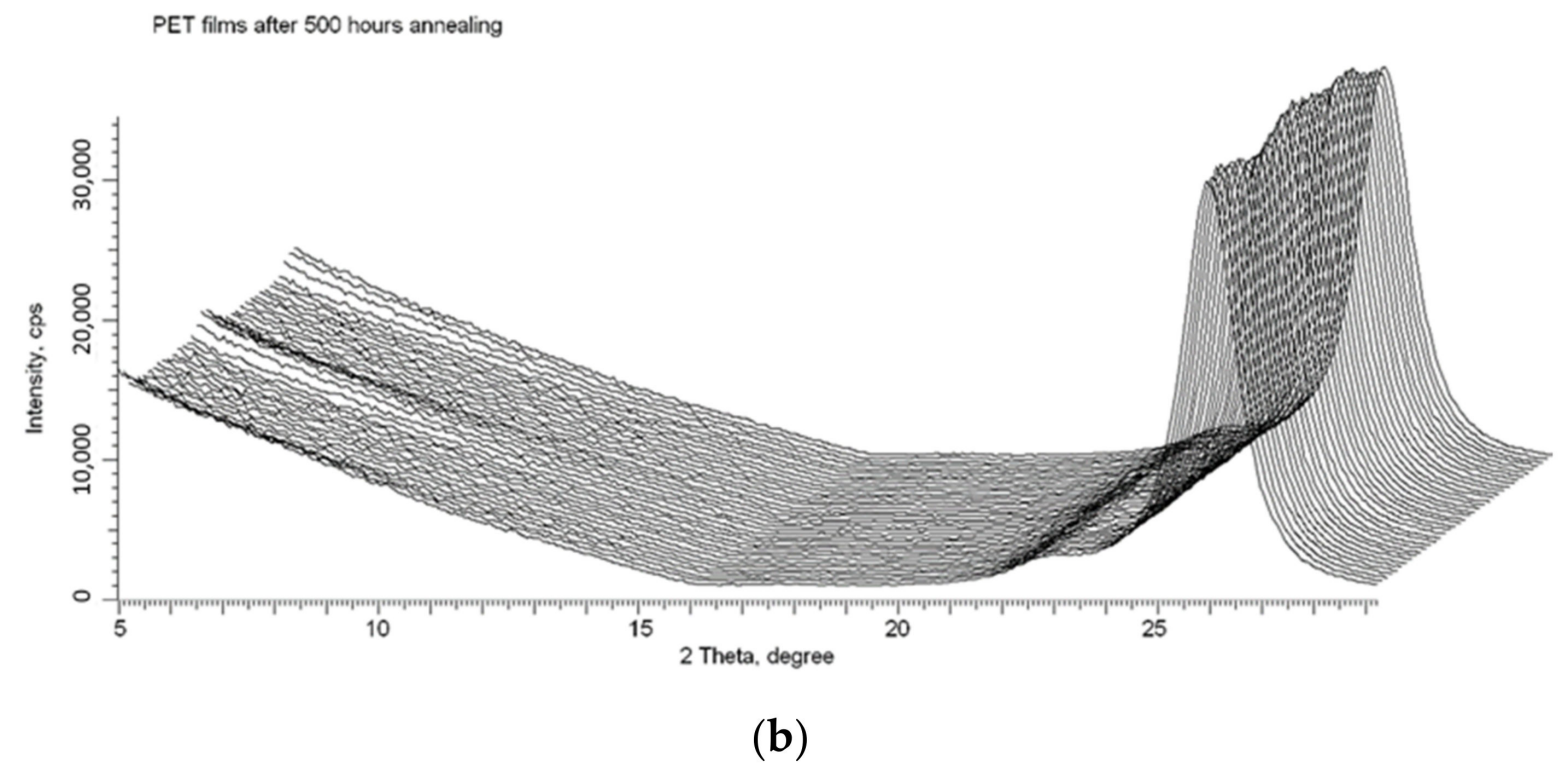
X-ray diffractograms in the geometry of *φ* = 0–2 *π* of the PET sample from Figure 9: (**a**) after irradiation; and (**b**) after 500 h annealing at a temperature of 75 °C.

We used the X-ray method described in detail in [16,17,18] to confirm that, despite the release of electrons from the core of the latent tracks to the peripheral zone, the spiral structures formed after irradiation remain stable. Figure 10a shows the X-ray diffractogram of the same PET film sample whose UV-vis transmission difference spectrum is shown in Figure 9, taken immediately after irradiation (by Kr^13+^ ions with an energy of 1.2 MeV/au at an angle of 40° and fluence of 5 × 10^10^ cm^−2^). As described above, following ion irradiation, the UV-vis spectrum was taken before and after the sample was annealed for 160 h. The sample was then returned to the annealing apparatus and annealed under the same conditions for a further 340 h (500 h in total). Figure 10b shows the X-ray diffractogram of the PET sample after this second period of annealing at a temperature close to the glass transition temperature. Comparison of these X-ray diffractograms clearly shows the persistence of signs of ordering [16] and spiralization [17] after prolonged annealing. This is good evidence of the stability of the intermolecular dipole–dipole interaction of benzene-carboxyl units in PET films after irradiation with swift heavy ions.

Finally, we consider whether, despite there being no immediately obvious connection, the red shift of the absorption edge in irradiated PET films reported in this article might be related to the post-irradiation ordering and spiralization of the molecular structure reported in [16,17,18].

**Figure 11:**
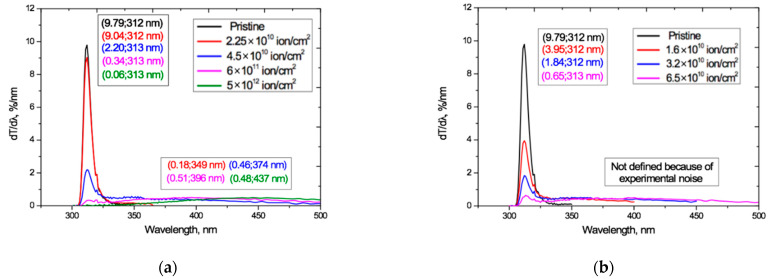
UV-vis transmission curve derivatives *dT_α_*(*λ*)/*dλ* derived from the transmission curves *T_α_*(*λ*) in Figure 4 for: (**a**) Ar^8+^ ions; and (**b**) Kr^15+^ ions.

We return to the interference-free UV-vis transmission curves *T_α_*(*λ*) shown in Figure 4. Figure 11 plots the first derivatives *dT_α_*(*λ*)/*dλ* of these curves, obtained by numerical differentiation (as was done in [19]). These derivatives give a clearer picture of the dynamics, due to the suppression by differentiation of the influence of slowly changing components in the original transmission functions. Differentiation of spectral functions has long been successfully used in modulation spectroscopy for detecting and studying weakly-expressed processes against backgrounds of general strong absorption. A classic example is that of resolving the 4.25 nm and 4.28 nm infrared doublet of a CO_2_ molecule by differentiating a wide and slowly changing absorption line [63].

The derivatives in Figure 11 show the presence of two modes that behave differently as the irradiation exposure increases, and reflect different processes (the small kink in all curves at 320 nm is an experimental artifact resulting from a change of illuminating bulb in our spectrophotometer).

For both Ar and Kr ion irradiation, the narrow peak seen in the pristine PET film, with a half-width in the wavelength range of 309–316 nm and a maximum at 312 nm, remains at the same position on the spectrum after irradiation, indicating the stability of the underlying physical process to irradiation. Taking into account the results of studies of the electret properties of PET films described above, we conclude that this peak is due to the photoactivity of the conjugated systems of benzene-carboxyl complexes in the repeat unit of chain molecules of PET [35]. The intensity of this narrow peak drops rapidly as the irradiation exposure increases, showing that the activity of these conjugated groups decreases as the irradiation fluence increases.

Figure 11 also shows the appearance under irradiation of a very broad flattened second peak that is not present in the pristine film. It extends over wavelengths beyond about 330 nm up to the observation limits of our apparatus and grows and shifts to longer wavelengths as the fluence increases. An explanation of this can be found in Britton’s studies of many natural pigments [64], including quinones, which have aromatic and carbonyl groups attached to them. Britton shows that the stability of conjugated systems increases with their increasing length and the consequent increased delocalization of electrons and lowering of the excitation energy, so that the peak of light absorption moves from the near-UV to the visible region as conjugated systems get larger. This effect is maximal for the coplanar conjugated systems. When the conjugated systems of neighboring molecules are sufficiently close, intermolecular electronic interactions can occur between them leading to the formation of various excited electronic energy levels. As in [49,50,51,52], Britton explains the colors of the pigments as being due to the absorption of light at wavelengths corresponding to complementary colors.

The well-known difference in the absorption edge of pristine PI and PET films described in our introduction is consistent with this explanation, since the conjugated system in PI is much larger than that in PET [29]. Furthermore, it has been shown by X-ray diffraction and quantum mechanical calculations that the only conjugated benzene-carboxyl unit of the repeat unit of the chain molecule of PET is stably coplanar, even in the monomer [65,66,67,68,69].

Figure 10 (above) shows the stability of both the post-irradiation intramolecular radial ordering of benzene-carboxyl units within the latent tracks and the intermolecular helical structures arising from this ordering as the fluence increases and the tracks begin to overlap [16,17]. The stability of these helical structures in irradiated PET films implies that the dipole–dipole bond between benzene-carboxyl units in neighboring PET chain molecules is stable and can be considered as a chemical bond, forming part of the well-known effect of cross-linking in irradiated films (see for example [25]). The formation of these helical structures leads to an increase in the length of extended conjugated bond systems, which as shown in [69] results in a red shift of the absorption edge.

We can now interpret the results in Figure 11 as follows. The decrease in the intensity of the sharp peak at 312 nm with increasing fluence is due to a decrease in the number of conjugated benzene-carboxyl complexes, as they progressively bond with others in the process of spiralization to form larger extended conjugated systems. At sufficiently high fluences, the peak is almost totally suppressed, which is in line with our suggestion in [18] that at high fluences it makes more sense to speak about a dipole–dipole cross-linked irradiated film, rather than the sum of the individual overlapping tracks.

## 5. Conclusions

By conducting a series of experiments irradiating PET films with different fluences and ion charges, we have shown that the total UV-vis optical response of irradiated PET films has a logarithmic dependence on the fluence. With appropriate calibration, this offers a new method of determining the irradiation exposure of PET films to swift heavy ions, which currently poses a technical challenge particularly at high fluences. We further demonstrate that this optical response has a dependency on the charge of the irradiating ion, providing independent confirmation of our results in [18] which also showed that the charge of the swift heavy irradiating ions has an effect on the irradiation-induced changes in PET films.

Previous experiments, at lower energies, have concluded that the behavior of the absorption edge red shift is due to carbonization of the polymer molecules. We show that, in the high ion energy regime used in our experiments, this explanation is insufficient. Our explanation is grounded in Bethe and Ashkin’s analysis showing that there are both fast (“delta”) and slow electrons involved in the interaction between swift heavy ions and the irradiated material. Drawing on well-established electret physics research and studies of the characteristics of aromatic and carbonyl chemical bonds, we present a physical interpretation of the observed absorption edge red shift as being due to the growth of extended conjugated systems through the formation of intermolecular helical structures in the irradiated PET film. Our investigations into the stability of irradiation-induced effects in PET films, though the comparison of UV-vis transmission spectra before and after annealing, shows that this technique can provide information about the structure of deep traps in PET.

## Figures and Tables

**Table 1:** Parameters for the ^84^Kr ion beam acceleration.

Ion	Energy, MeV/au	Energy, MeV	Field, T	HF, MHz
^84^Kr^15+^	1.1905	100	1.25015	13.712
^84^Kr^14+^			1.33943	13.712
^84^Kr^13+^			1.4425	13.713

## Data Availability

Not applicable.
